# Functional differences of *Porphyromonas gingivalis* *Fimbriae* in determining periodontal disease pathogenesis: a literature review

**Published:** 2013-03-30

**Authors:** Sandra Moreno, Adolfo Contreras

**Affiliations:** a Escuela de Odontología de la Facultad de Salud de la Universidad del Valle, E-mail:odsandramoreno@hotmail.com; b Grupo de Investigación Medicina Periodontal de la Universidad del Valle, E-mail:adolfoco@yahoo.com

**Keywords:** *Porphyromonas gingivalis*, bacterial adhesion, periodontitis, *Fimbria*e, *FimA*, genotipification

## Abstract

P*orphyromonas gingivalis *is implicated in chronic and aggressive periodontitis. This bacterium has numerous virulence factors and one is the *Fimbria*e, which is quite important for bacterial colonization. *Fimbriae* are appendices that anchor to the bacterial wall and are comprised of the protein *FimB*riline encoded by the *FimA* gene. Thus far, six genotypes have been identified, *FimA* I to V and Ib. Genotypes II and IV are associated with periodontal disease, while genotype I is related to gingival health. Genotype identification of *P. gingivalis* *FimA* in periodontitis would be important to confirm the pathogenic genotypes and to establish risk at population level. This review is about the *P. gingivalis* *FimA* genotype prevalence worldwide. A systematic search using Pubmed, Hinary, and Science Direct within the following descriptors: *Porphyromonas gingivalis*, bacterial adhesion, periodontitis, *Fimbria*e, *FimA*, genotipification was performed to April 2011.

## Abstract (es)

P*orphyromonas gingivalis* es un microorganismo implicado en la periodontitis crónica y agresiva. Dentro de sus factores de virulencia, se encuentran las *Fimbria*s, las cuales están compuestas por una proteína denominada *FimB*rillina, que está codificada por el gen *FimA*, del cual existen 6 genotipos (I, II, III, IV, V, Ib), según la secuencia de nucleótidos. Los genotipos II y IV han sido relacionados con periodontitis, mientras el I con salud periodontal. Identificar los genotipos de *FimA* de *P. gingivalis* en pacientes con periodontitis podría generar nuevas estrategias que conlleven a suprimir los genotipos más patogénicos para prevenir el desarrollo de la periodontitis en portadores sanos. Se revisó la prevalencia de los genotipos de *FimA* de *P. gingivalis* en diferentes países del mundo, para lo cual se realizó una búsqueda sistemática en bases de datos de Pubmed, Hinary y Science Direct usando los descriptores: *Porphyromonas gingivalis*, adhesión bacteriana, periodontitis, *Fimbria*s, *Fim A*, y genotipificación hasta abril del 2011.

## Introduction

Periodontitis is an inflammatory disease that affects the protective connective tissue, the sulcular epithelium, and the supporting tissue of the teeth like the periodontal ligament and the alveolar bone^1^ ^,^ ^2^.

One of the disease's triggering factors is the persistence of the biofilm formed over the dental surfaces and in the subgingival environment^3^, which stimulates the immune response of the host and induces the production of proinflammatory cytokines and lysosomal enzyme release by macrophages, besides reduced collagen synthesis by fibroblasts and increased metalloproteinases of connective tissue with concomitant reduction of their inhibitors. This unbalance starts the destruction of periodontal tissue, including bone resorption due to the chronicity of the microbial and inflammatory challenge^4^.

Diverse microorganisms have been implied in the pathogenesis of periodontitis, like: *Porphyromonas gingivalis, Prevotella intermedia, Tannerella forsythia, Fusobacterium nucleatum, Aggregatibacter actinomycetemcomitans, Micromonas micra, Eikenella corrodens, Treponema denticola,*and *Campylobacter rectus*, among other species^5^.

These microorganisms must find an appropriate ecological niche or activity site in the host to establish themselves and start to grow and multiply, in such a way that they penetrate the physical barriers like the junctional and surcular epithelium, degrade the immunoglobulins in the crevicular fluid and in the saliva, and survive the local action of polymorphonuclear leukocytes (PMN), to then associate with the epithelial cells, cells of the basal layer of the epithelium, and probably the fibroblasts. This association or bacterial adherence, essential for colonization and pathogenicity, is established through the virulence factors that like the *Fimbriae* facilitate colonization, maintenance, and protection of the bacterial species in the host^6^.

For *Porphyromonas gingivalis (P. gingivalis)*, the virulence factors are: the capsule^7^, proteins of the outer membrane, lipopolysaccharides^6^, proteases like gingipains, collagenases, hemolysin, trypsin proteases, hemagglutinins^8^, and *Fimbria*e, which are involved in colonization, invasion, establishment, and persistence within the host, evasion by the destruction of the mechanisms of the immune system and damage to protective periodontal tissues^6^ ^,^ ^7^.

This literature review (Pubmed, Hinari and Science Direct to April 2011) emphasizes on one of the virulence factors of *Porphyromonas gingivalis* such as *Fimbriae* encoded by the *FimA* gene, its prevalence in different world populations and the clinical importance from its distribution in the stages of periodontal disease. A systematic search was conducted using the health descriptors: *Porphyromonas gingivalis,* *FimA*, genotipification, colonization, and periodontitis in Pubmed, Hinary, and Science Direct databases.

## Porphyromonas gingivalis

*P. gingivalis* is an anaerobic, non-motile, Gram negative coccobacillus, which is related to the start and progression of the chronic and aggressive periodontal disease; it can be considered one of the main etiologic agents of destructive periodontal disease^9^ ^,^ ^10^, although it is a microorganism that has also been isolated in gingivitis and in healthy patients in low proportions^7^.

Likewise, it has been implied in diverse systemic complications like cardiovascular disease, preeclampsia, and low birth weight^10^ ^,^ ^11^, given its capacity to colonize other tissues, which has been evidenced by its presence in atheromatous plaques^1^.

According to the model of the dental biofilm complexes proposed by Socransky and Hafajee, *P. gingivalis* belongs to the red complex; hence, it is part of the tertiary colonizers that colonize dental and periodontal tissue when the biofilm is mature^3^.

This colonization is influenced by saliva, which functions as a vector for its transmission and entry into the oral environment; additionally, the film acquired of the saliva on the dental surface facilitates adhesion of the bacteria *Fimbria*e, which bond to solid surfaces with high affinity hindering its removal by the salivary flow^1^. Finally, these are saccharolytic bacteria with proteolytic activity.

**Figure 1:**
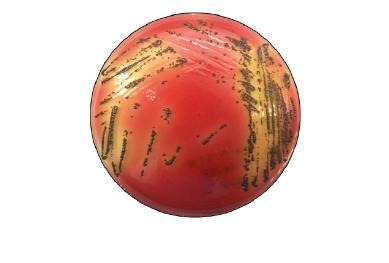
Colony of P. gingivalis, W83 strain, cultivated in blood agar. These are observed in black due to their capacity to attract iron from the culture medium.

*In Vitro*, when growing in agar supplemented with sheep blood, hemin and menadione in incubation in anaerobiosis for 10 to 15 days, the colonies are initially observed as yellow or brown colored and after 4 to 8 days they darken from the border to the center until turning black due to their capacity to attract iron from the culture medium^12^ (Figure 1). 

### Fimbriae from Porphyromonas gingivalis

Among the virulence factors that develop *p. gingivalis*, there are *Fimbria*e, which have been considered the main virulence factor of this microorganism, given that it confers it the capacity to adhere and invade tissues, which characterizes its high pathogenicity over periodontal tissue^9^.

*Fimbriae* are fine and numerous appendices that protrude from the outer cell membrane, whose main function is adhesion to periodontal tissue^12^, endothelial cells^1^, and other tissues^8^, given that they have isolated from ovarian and lung abscesses^10^, and it has even been found that *P. gingivalis* *Fimbriae* mediate the congregation with *Streptococcus oralis* through molecules, residues, or specific domains^13^.

Two types of *Fimbriae* exist, the 41-KDa (major) determined by Yoshimura *et al*., in 1984, and the 67-KDa (minor) found by Hamada *et al*., in 1996^14^. The major, which configure long appendices measuring approximately 0.3 to 1.6 micra long^15^, is comprised of subunits of a protein called *FimB*riline, which is encoded by a gene denominated *FimA* ^14^ of which only one copy exists in the *P. gingivalis* ^16^ chromosome. Minor *Fimbriae* are comprised of minor *Fimbria* protein subunits (Mfa1) encoded by the mfa1 gene^14^; these *Fimbriae* measure from 3.5 to 6.5 nanometers long, significantly shorter than the major *Fimbria*e^15^.

In research on mice, it has been found that *Fimbriae* stimulate the production of interleukin 1 (IL-1), interleukin 6 (IL-6), interleukin 8 (IL-8), and tumor necrosis factor (TNF) by peritoneal macrophages and that in humans it triggers TNF secretion per monocyte^6^. These cytokines are potent inflammatory mediators, which can lead to the activation and destruction of bone tissue and periodontal tissue. Hence, *Fimbria*e, besides being considered important virulence factors in colonization and invasion of *P. gingivalis* in oral tissues, can also collaborate with the inflammatory response dependent on the immune response upon stimulating secretion of the cytokines mentioned.

Studies in experimental animals have shown that immunization with *P. gingivalis* *FimB*riline protects the individual from destruction of periodontal tissue, but due to the genotypic diversity observed in this bacteria structural and antigenic heterogeneity exists of the *Fimbria*e, in such a manner that if antibodies are developed against a specific type of *Fimbriae* protection is not accomplished against the other types^17^.

### Genotypic and phenotypic variety of the Fimbriae

*P. gingivalis* is a microorganism with considerable genotypic diversity; hence, we can find clones more pathogenic than others and this could be the reason that explains the presence of the bacteria in healthy patients who have no signs of periodontal disease and in patients with severe periodontal disease, where there are signs of marked destruction of supporting tissue.

*FimA* is the gene that encodes the *Fimbriline* subunits. Until now, six *FimA* genotypes have been found (I, Ib, II, III, IV, V) based on their nucleotide sequence.^8^ ^,^ ^12^ *FimA* *Fimbriae* adhere to different proteins of eukaryotic cells like fibronectin, collagen, laminin, the proline-rich protein derived from saliva and statherin, as well as to prokaryotic proteins like glyceraldehyde-3-phosphate dehydrogenase (GAPDH) of* Streptococcus oralis.*

The *FimA* gene is found in a gene cluster that encode for regulatory factors or *Fimbriline* accessory proteins. Downstream from *FimA* there are four genes denominated *FimB*, *FimC*, *FimD*, and *FimE*. Recently, it was described that *FimC*, *FimD*, and *FimE* products playa n important role in *Fimbria*e, in that if they are suppressed in *in vitro* experiments, the ability of *P. gingivalis* to adhere to eukaryotic and prokaryotic cells, which affects the capacity of *P. gingivalis* *Fimbriae* to colonize tissues, affecting autoaggregation^15^.

Nagano *et al*., in 2010^18^, conducted a study with the *FimB* gene and concluded that this gene regulates *FimA* length and expression, which is important for colonization and adhesion of the microorganism and generated the hypothesis that the *FimB* gene would be implied in producing an element responsible for the anchoring of *Fimbriae* to the bacterial outer membrane.

## Prevalence of FimA in diferent world populations

**Figure 2:**
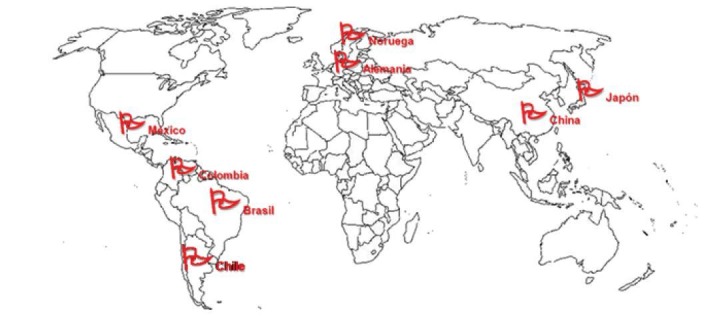
Geographic distribution of world populations where studies have been done on Genotipification of the fimA gene of Porphyromonas gingivalis.

Genotypic variability among *P. gingivalis* isolates is a reflection of the distinct sequences of its DNA, for this reason the molecular tipification methods have been a big contribution for its study^19^. In specialized literature, it is possible to find various studies that have determined *FimA* prevalence in different world populations (Figure 2).

Because of this, a literature review must be conducted; a dendrogram was carried out in this manuscript to determine population differences through the geographic distribution of said gene, in *P. gingivalis* (Table 1).

**Table 1:**
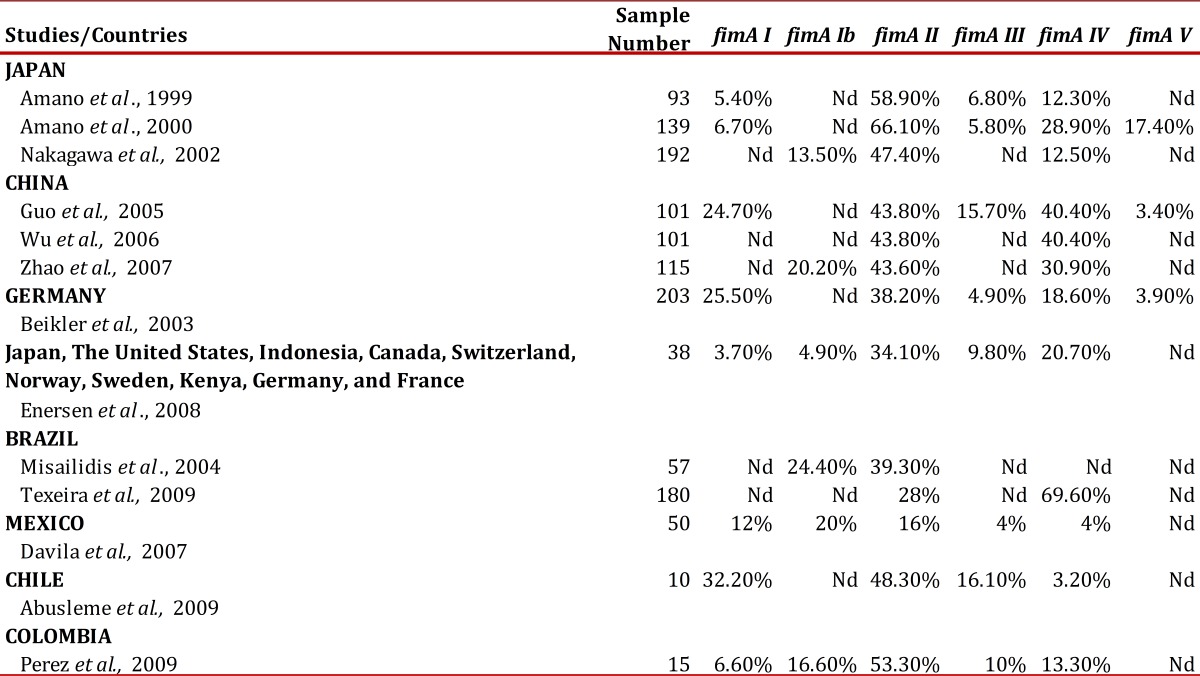
Frequencies of Porphyromonas gingivalis fimA genotypes in patients with periodontitis in different world populations.

In Japan, Amano *et al*., have done extensive research of *P. gingivalis* *Fimbriae* and their distribution and prevalence in that country's population. In a study in 1999,7 they found that *FimA* II followed by *FimA* IV are the most prevalent genotypes in patients with periodontal disease. In that study, they selected 93 Japanese adults with periodontal disease and without systemic involvement (33 men between 19 and 74 years of age and 60 women between 21 and 79 years of age). It should be mentioned that in 1999 only four 4 *FimA* genotypes were known (from I to IV). The results demonstrated that of 93 patients, 73 were positive for *P. gingivalis*, of which *FimA* type II was the most prevalent with 58.9%, compared to 5.4%, 6.8%, and 12.3% for type I, III, and IV, respectively. They also observed a relationship between *FimA* II with the depth of the pocket, given that in the deepest 90.9% was found, compared to the other genotypes studied, and that in women aged between 36 and 57 years the highest percentage of 83.3% was found for *FimA* II. Thereby, Amano *et al*., 1999, concluded that the *FimA* II genotype is most prevalent in patients with periodontal pathology and that the probability exists of other genotypes different from genotypes from I to IV, which is why they recommend conducting further studies.

In 2000, Amano *et al*.^20^ managed to clone a new *FimA* genotype, the type V. Given that in prior studies some patients who were positive for *P. gingivalis* had none of the four genotypes known till then; it was suspected that another non−described genotype existed. Of the 73 samples used in the previous study, five specimens had the non-described genotype; hence, these samples were used as templates to amplify the new *FimA* gene by using a PCR test. The PCR products were separated via electrophoresis and the DNA was cloned in a vector (*E. coli*).

Later, in 2000, Amano *et al*.^21^ studied 380 periodontally and systemically healthy Japanese adults (163 men between 30 and 70 years of age − mean of 51.23 ± 11.2 years of age − and 217 women between 30 and 70 years of age) and 139 Japanese adults with periodontitis and without systemic involvement (70 men between 30 and 70 years of age and 69 women between 30 and 70 years of age) in which samples were obtained to genotype *P. gingivalis* via PCR. Of 380 periodontally healthy patients, *P. gingivalis* was identified in 138 (36.3%), while of 139 patients with periodontitis it was identified in 121 (87.1%). In periodontally healthy subjects, *FimA* type I was observed in 76.1%, type II in 9.4%, type III in 7.2%, type IV in 6.5%, and type V in 29.7%; while in subjects with periodontitis, *FimA* type I was observed in 6.7%, type II in 66.1%, type III in 5.8%, type IV in 28.9%, and type V in 17.4%. From this, we can conclude that in periodontally healthy patients types I and V are most prevalent (*p *<0.00001 and *p *<0.0001, respectively), while types II and IV were most prevalent in patients with periodontitis (*p *<0.00001).

In 2002, Nakagawa *et al*.^9^cloned a new *FimA* genotype, which was designated *FimA* Ib, indicating that it is a variant of the *FimA* gene I, given that the 97.1% homology between both genotypes. The researchers used a sample of 380 periodontally and systemically healthy patients (men and women aged between 30 and 70 years) and 192 patients with periodontal disease and without systemic involvement (men and women aged between 30 and 70 years). Additionally, they analyzed samples of 44 adult patients with trisomy 21 (men and women aged between 20 and 35 years) and 39 adults with mental retardation (men and women aged between 20 and 35 years). With the samples of these last patients genotipification was done with the six genotypes described and it was found that in patients with periodontitis the type II (47.4%), type Ib (13.5%), and type IV (12.5%) genotypes are relatively more prevalent compared to healthy patients, who presented frequencies of 50.4% in type I and 12.2% in type V. Likewise, this study reported that the odds ratio (OR) for the adult population in Japan (indicating the relationship between periodontitis and *FimA*) was higher for *FimA* II (OR 77.80 with 95% confidence interval of 31.0 - 195.4).

Several studies have reported the relationship between periodontal disease and cardiovascular disease, while at the same time the presence of *P. gingivalis* was found was found in specimens of atheromatous plaques. Nakano *et al.* ^1^ demonstrated the presence of *P. gingivalis* bacterial DNA in 35 cardiovascular specimens collected during surgery of 35 heart valves. In 11.4% *P. gingivalis* DNA was detected, while of 27 specimens of atheromatous plaques, *P. gingivalis* DNA was detected in 7.4%. However, the same authors posed the objective of confirming the prevalence and distribution of *FimA* genotypes in these specimens for which they collected cardiovascular specimens during 2.5 years (between December 2004 and May 2007). These specimens consisted of 112 excised heart valves, of which 14 were through diagnosis of infective endocarditis and 98 through non-infective endocarditis, in addition to 80 specimens of atheromatous plaques with diagnosis of abdominal or thoracic aneurism, for a total of 192 specimens. From 54 of these patients who were subjected to surgery samples were taken of bacterial plaque. The results revealed that of 192 cardiovascular specimens, 20 were positive for P.gingivalis and of 56 samples of bacterial plaque, 28 were positive for *P. gingivalis*. In the cardiovascular samples the most frequent genotypes were *FimA* II (30% -6 specimens) and *FimA* IV (45% - 9 specimens) and of the samples of plaque higher prevalence was found in genotypes II (35.7%), I (28.6%), and IV (21.4%). Finally, the authors concluded that the *FimA* genotypes that have been associated to periodontitis are also frequently found in cardiovascular specimens, which suggests the possible role of the type II and IV clones at the start and progression of cardiovascular disease; although due to the low amount of cardiovascular samples positive for *P. gingivalis* more studies are required to confirm this hypothesis.

In 2004, in an experimental study conducted by Nakano *et al*. cited by Hajishengallis in 2009^22^, it was found that *FimA* A II and IV genotypes were more aggressive and generated greater damage to the tissue than *FimA* I genotypes in a model of systemic infection disseminated in mice.

Other authors in Japan have also studied the prevalence and distribution of *FimA* genotypes. Miura *et al* ^23^. in 2005, analyzed the prevalence of *FimA* genotypes in patients with aggressive periodontitis. In that study, they included 18 patients with aggressive periodontitis and 22 periodontally healthy patients. In the patients with aggressive periodontitis, the most prevalent genotype was *FimA* II (40.5%), followed by *FimA* Ib (23.8%), and *FimA* I (23%). The only genotype found in healthy patients was the *FimA* I genotype with 100% frequency. These results are different from those reported in other studies in which the *FimA* IV genotype is the second most prevalent and the *FimA* I genotype is characteristic of healthy patients; however, in this research the genotipification was done specifically on patients with aggressive periodontitis and a variation can exist in P. gingivalis genotypes with respect to the other types of periodontitis, which could change the course and evolution of the pathology.

*Guo et al*. 2005^24^ in China, investigated the distribution of *P. gingivalis* *FimA* genotypes in samples of subgingival plaque from 101 patients with chronic periodontitis. The distribution and prevalence of *FimA* genotypes was conducted via PCR with specific primers for each. *P. gingivalis* was found in 88.1% of the samples and genotype distribution was as follows: type I, 24.7%; type II, 43.8%; type III, 15.7%; type IV, 40.4%; and type V, 3.4%. It is concluded that *P. gingivalis* was positive in most of the subgingival samples of the patients with chronic periodontitis, and the *FimA* II and *FimA* IV genotypes were the most predominant in these patients; hence, these are the genotypes that are most associated to the development of periodontitis.

*Wu et al*. in 2006^25^ in the School of Stomatology at the University of Sichuan in west China, analyzed the distribution of *Porphyromonas gingivalis* *FimA* genotypes in Chinese patients with periodontitis and their relationship between *FimA* genotypes and chronic periodontitis. Subgingival samples were collected from 101 patients with periodontitis. *P. gingivalis* was detected in 89 patients (88.1%) and the most frequent genotype was *FimA* II (43.8%) and *FimA* IV (40.4%). These two genotypes were also most prevalent in patients with moderate and severe periodontitis. The authors concluded that *FimA* II and *FimA* IV genotypes are the most prevalent in patients with periodontitis in China and these genotypes are involved in the progression of periodontal destruction.

*Zhao et al*. in 2007^26^, analyzed the prevalence of *FimA* genotypes in 115 adult patients with chronic periodontitis in China, (67 men and 48 women between 25 and 75 years of age) and 136 periodontally healthy adult patients (44 men and 92 women between 25 and 75 years of age). Thirty of 136 healthy patients (22.1%) and 94 of 115 patients with periodontitis (85.1%) were positive for P. gingivalis. In addition, the authors found that in healthy patients 62.7% had the *FimA* I genotype, 16.7% *FimA* V genotype, and 10% *FimA* II genotype I; while in patients with chronic periodontitis the most prevalent genotypes were *FimA* II (43.6%), followed by *FimA* IV (30.9%) and *FimA* Ib (20.2%). Likewise, the study correlated the prevalence of genotypes with pocket depth, so that the most prevalent genotypes in pockets bigger or equal to 7 mm were *FimA* II (55.3%) and *FimA* IV (30.9%), while the *FimA* V genotype was the least prevalent in deep pockets (4.3%). Also correlated was the prevalence of *FimA* with bleeding upon probing, with *FimA* II (45.8%) and *FimA* IV (32.5%) genotypes being the most prevalent, and the least prevalent being the *FimA* V genotype (3.6%). Finally, this study also analyzed the relationship between *P. gingivalis* *FimA* genotypes and their association to other bacteria like *Tannerella forsythia* and *Aggregatibacter actinomycetemcomitans;* so that of 30 healthy patients positive for *P. gingivalis,* six were positive for *T. forsythia* and one for *A. actinomycetemcomitans,* while of 94 patients with chronic periodontitis positive for *P. gingivalis*, 60 were positive for *T. forsythia* and 34 for A. *actinomycetemcomitans*. Upon correlating the genotypes, the authors found that the frequency of coexistence of *T. forsythia* with *FimA* II was of 53.3% and the frequency of coexistence of *FimA* II with *A. actinomycetemcomitans* was 44.1%, with this genotype being the most frequently associated to other bacterial species that colonize the periodontium. Similarly, this study reported that the OR for the Chinese population regarding the relationship between the *P. gingivalis* *FimA* genotype and periodontitis was: for type I (0.97), type Ib (13.26), type II (36.62), type III (4.57), type IV (22.86), and type V (1.19), being the highest for the *FimA* II type, as in Japan.

In Germany, Beikler *et al*. 2003^27^, conducted a study on the prevalence of *FimA* genotypes in 102 Caucasian patients (73 women and 29 men). The researchers initially took a universe of 203 patients of which 102 were positive for *P. gingivalis* (50.25%) and in whom the study was finally conducted. The research reported that 26 patients (25.5%) had *FimA* I, 40 patients (38.2%) had *FimA* II, 5 patients (4.9%) had *FimA* III, 19 patients (18.6%) had *FimA* IV and 4 patients (3.9%) had *FimA* V genotype. According to the authors, the differences with the results in Japan are attributed to the adaptation of the genotypes to environmental changes.

Also, Enersen *et al*. in 2008^16^, carried out a study of *FimA* prevalence in 38 samples of patients with periodontitis from various countries like Japan, the United States, Indonesia, Canada, Switzerland, Norway, Sweden, Kenya, Germany, and France;^28^ isolates from Finland and 13 isolates from Holland. The distribution of the genotypes was *FimA* II (34.1%), *FimA* IV (20.7%), *FimA* III (9.8%), *FimA* Ib (4.9%), and *FimA* I (3.7%). The *FimA* V genotype was only detected in one isolate.

For Latin America, few studies have been reported. In Brazil, Misailidis *et al*. 2004^29^, analyzed 102 patients (men and women between 14 and 75 years of age, from a multiethnic Brazilian population with different periodontal conditions (healthy patients, patients with gingivitis, and patients with chronic and aggressive periodontitis). Initially, the presence of *P. gingivalis* was confirmed in the samples, which was positive in 2 of 25 healthy patients, 6 of 20 patients with gingivitis, 38 of 42 patients with chronic periodontitis, and 13 of 15 patients with aggressive periodontitis. The genotype distribution for this population was for patients with periodontitis; the most prevalent genotype was *FimA* II with 39.3%, followed by *FimA* Ib with 24.4%. In healthy patients, only one sample was positive for *FimA* II and in patients with gingivitis only two samples were positive for *FimA* IV. These results contrast with those reported in Japan and China, where, after *FimA* II the most prevalent is *FimA* IV, according to the authors this could be due to the racial heterogeneity of the Brazilian population and to the difference of its origins, confirming the variability of the population. This study mentions that a non-specific *FimA* genotype was found in 17% of the subjects studied, different from the 5.1% reported by researchers in Japan, which suggests the existence of other genotypes that have not been studied. It is concluded that the most prevalent genotype in patients with periodontitis is *FimA* II and that it must be considered as the most virulent and pathogenic, given that it has the greatest capacity for adhesion and invasion of tissues and that the majority of smokers had periodontitis and were positive for *P. gingivalis.*

Texeira *et al*. in 2009^12^, conducted a study about the distribution of *FimA* genotypes in Brazilian patients who smoked and had chronic periodontitis, which quantitatively evaluated the levels of *FimA* II and *FimA* IV genotypes and in patients who smoked and had chronic periodontitis via reverse transcription polymerase chain reaction (RT- PCR). They found that *FimA* II was positive in 18 of 20 subjects and that *FimA* IV was found in the 20 subjects. Samples were collected from nine sites from each patient, for a total of 180 sites of which 90.5%, i.e., 152 sites were positive for *P. gingivalis*, of these 28% of the sites had *FimA* II; in contrast with *FimA* IV that was found in 69.6% of the sites. Also analyzed was the frequency of *FimA* II and *FimA* IV genotypes in different categories of pocket depth, observing that in this population the *FimA* IV genotype is more frequent in the deepest periodontal pockets. These results suggest that *FimA* IV is related to deeper pockets in patients with chronic periodontitis and that it is the most prevalent genotype in Brazilian patients who smoked and had periodontitis, suggesting that in this population it is the most virulent genotype.

Davila *et al*. in 2007^30^, analyzed the distribution of the *P. gingivalis* *FimA* gene in patients with Diabetes type II and with periodontitis in Mexico. A total of 75 patients were selected, divided into three groups; group 1 comprised 25 healthy, non-diabetic, patients (8 men and 17 women aged between 30 and 62 years); group 2 comprised non-diabetic patients with chronic periodontitis (5 men, 20 women aged between 30 and 70 years); group 3 comprised patients with diabetes mellitus type II and with chronic periodontitis (13 men, 12 women, aged between 37 and 68 years). Subgingival samples were taken with sterile Gracey curettes, prior removal of supragingival plaque, from the distal-lingual surface of the mandibular left lateral in all the patients. DNA extraction and genotipification were carried out via PCR, using specific primers for each *FimA* genotype. In group 3, five patients were positive for *P. gingivalis* as of gene 16S rRNA, but negative for the specific primers of the *FimA* genotypes. These products were amplified with primers for *P. gingivalis* 16S rRNA and cloned in a vector (pGEM T-Easy vector(r)) and delivered for analysis in the Research Institute in Irapuato, Mexico (CINVESTAV-IPN). *P. gingivalis* was positive in group 1 (38%), group 2 (70%), and group 3 (70%). These five clones not amplified with *FimA* primers but with the *P. gingivalis* 16S rRNA universal primer had a close phylogenetic relationship with the *FimA* I genotype, which was analyzed and observed through a distance matrix. In all the groups, the most prevalent genotypes were type I, Ib, and II, with differences in distribution in the different groups. In group 1, the most prevalent genotype was *FimA* I (40%); individually and in association with other genotypes, the most prevalent was also *FimA* I; in group 2, *FimA* Ib was observed in greater proportion (20%); individually and in association it was found in 36%; in group 3, the genotypes individually found most often were I (20%) and III (20%).

A difference is noted with the high prevalence of *FimA* I and III genotypes in patients with diabetes type II and periodontitis. In studies in Brazil and Japan the genotype most associated to periodontitis is type II, but periodontal disease in diabetics can also be attributed to other factors that can be altered in the innate immunity, alterations in microvasculature and final metabolite glycolysis.

In Chile in 2009, Abusleme *et al*. genotyped^31^ *P. gingivalis* isolates obtained from a group of 10 Chilean patients, (seven with chronic periodontitis and three with aggressive periodontitis), these isolates mostly presented the *FimA* II genotype (48.3%), followed by *FimA* I (32.2%). The lowest percentages were the *FimA* III (16.1%) and *FimA* IV (3.2%) genotypes; additionally, the study reported the presence of more than one type of *Fimbriae* in some patients.

In Colombia in 2009, Pérez *et al* ^31^. conducted an interesting study on *FimA* genotypes. They selected 15 patients, nine with chronic periodontitis and six with aggressive periodontitis who presented pockets > 7 mm and were candidates for periodontal surgery. Samples of subgingival plaques were taken prior to surgery along with a blood sample during the bacteremia after the scaling and root planing. Four blood samples were taken per patient; the first before the procedure; the second immediately at the end; the third, 15 minutes after; and the fourth, 30 minutes later. The surgical procedure was performed during 10 minutes (10 sites per patient, one minute per site). The technique used for genotipification was PCR. Six patients resulted positive via bacteremia for P. gingivalis; this finding confirms this pathogen's capacity to enter the circulatory system.

In 30 subgingival-plaque isolates a higher prevalence of *FimA* II was found, followed by *FimA* Ib, *FimA* III, and *FimA* IV. In blood isolates the most prevalent genotypes were *FimA* II followed by IV types, Ib and III. The most prevalent genotypes in these patients, both in the sample from subgingival plaque and strains found in blood, was *FimA* II, which agrees with that found by Enersen *et al*. in 2008^16^, Van der Ploeg *et al*., in 2004^27^, Beikler *et al*., 2003^26^, Misailidis *et al*., in 2004^28^, Amano *et al.,* 2000^20^, and Zhao *et al*., in 2007^25^. In general, the results from these studies have shown that the *FimA* gene II is the most frequent in patients with periodontitis followed by *FimA* IV, while in healthy patients the most frequently found is the *FimA* genes I and III; however, certain differences have been found among the different populations and this has been attributed to ethnic differences existing in populations like Brazil^12^ ^,^ ^29^, Mexico^30^, and Chile.

The *FimA* I genotypes are less aggressive; they are associated to the first stages of the infection like colonization, invasion, and subversion of the immune response, besides lacking a capsule, which makes them less virulent bacterial strains with low invasion capacity in tissues; in contrast, *FimA* II and *FimA* IV genotypes are considered pro-inflammatory genotypes, which exhibit a more aggressive phenotype with capacity to cause damage to the tissue; generally, these genotypes are encapsulated and this gives them an advantage in terms of invasion, survival within the host, and resistance, which is related to the chronicity of the infection^7^ ^,^ ^21^. It has been found, in experimental studies that *FimA* II can adhere to epithelial cells and invade the cell more efficiently than the other genotypes and it does it through specific receptors in the host, including Integrin ∞5ß1^14^ ^,^ ^15^.

## Biological distances of porphyromonas gingivalis FimA ii and FimA iv genotypes

Various studies reported differences in the distribution of the *Porphyromonas gingivalis* *FimA* genotypes^26^ ^,^ ^28^ (Table 2), which were attributed to environmental factors, racial heterogeneity of the population, and systemic diseases like diabetes that alter the immune system.

**Table 2:**
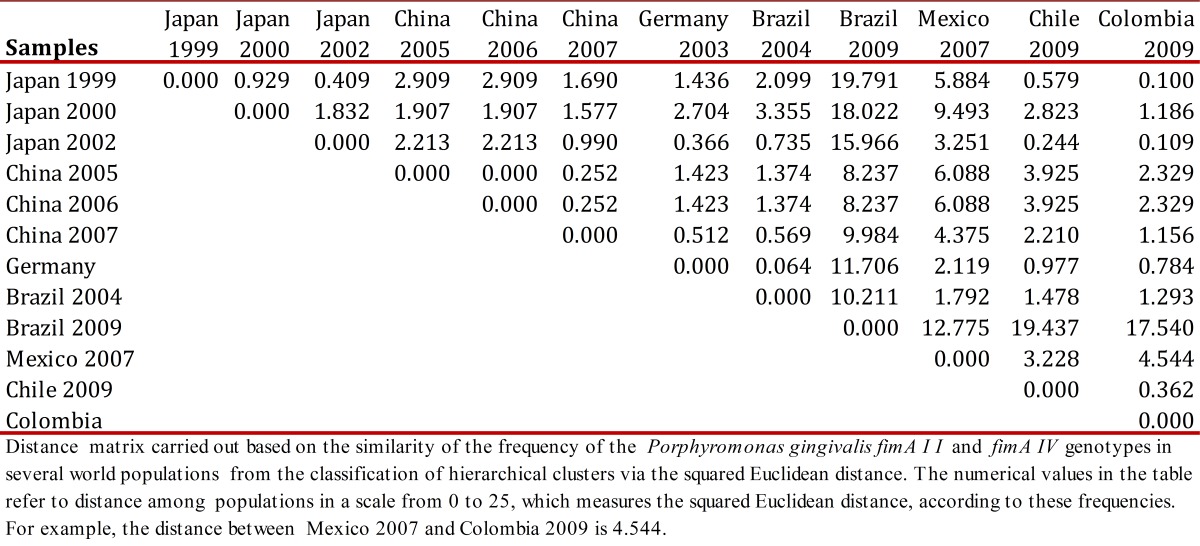
Distance matrix between the frequency of fimA II and fimA IV among world populations based on the squared Euclidean distance .

**Figure 3:**
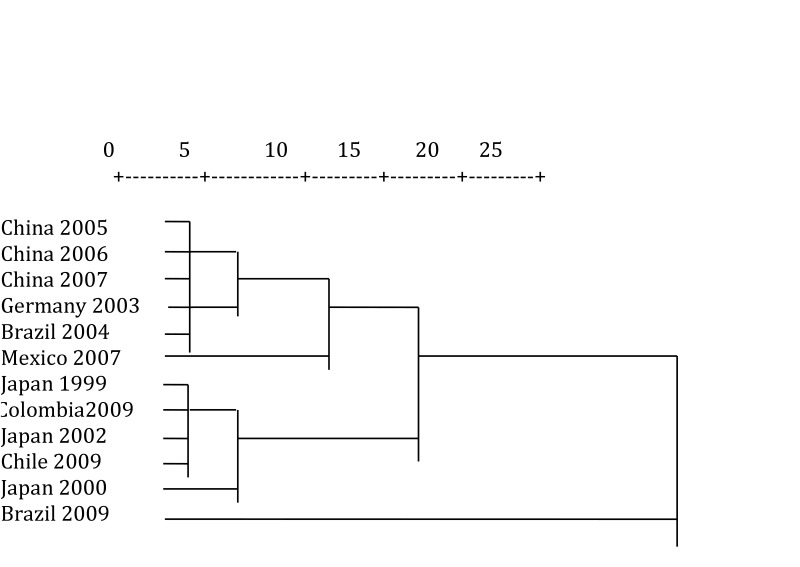
Dendrogram, using Ward's method, derived from the distance matrix between frequency of fimA II and fimA IV of world populations based on the squared Euclidean distance. The scale above goes from 0 to 25 to measure the squared Euclidean distance.

Due to this, this report used the SPSS software ver. 17.0 in English to create a distance matrix (Table 2) based on the frequency similarity of *FimA* II and *FimA* IV in several world populations from the classification of hierarchical clusters through the squared Euclidean distance. In addition, the respective dendrogram was obtained (Figure 3) by using Ward's method^32^ in which the elements to be related are clustered hierarchically, according to their similarity to evidence the levels of proximity among the populations included in the matrix.

The dendrogram shows the populations considered in the studies in China 2005^23^, China 2006^25^, and China 2007^26^ are part of the first cluster from the frequency of *FimA* II and *FimA* IV, which indicates that the distribution of these genotypes is similar in these studies. Germany 2003^27^ and Brazil 2004^29^ make up the second cluster; Mexico 2007^30^ is part of the third cluster; Japan 1999^7^, Colombia 2009^31^, Japan 2002^9^, and Chile 2009 comprise the fourth cluster; Japan 2000^20^ the fifth and Brazil 2009^12^ the sixth cluster. These groupings into clusters show the affinity in the results of the studies conducted in the countries that are included in each of the six previously mentioned.

This rather heterogeneous distribution of the populations, bearing in mind their origin and geographic location, can be associated to the number of samples, for example, the 1999^7^study by Amano *et al.,* took 93 patients with chronic periodontitis, which differs from a later study by Amano *et al.,* in 2000^20^, which took 380 healthy patients and 139 patients with periodontitis; this is also contrasted with studies in China, where, while Guo *et al.,* in 2005^24^ took a sample of 101 patients with periodontitis, Zhao *et al.,* in 2007^26^ worked with 115 patients with periodontitis and 136 healthy patients. This also differed with the study in Germany by Beikler *et al.,* in 2003^27^, who used a sample of 203 patients. In Brazil, both studies reported also present big differences in their sample number, Missailidis *et al.,* in 2004^29^ worked with 102 patients with periodontitis, while Texeira *et al.,* in 2008^12^ took a sample of 20 smokers with periodontitis.

In Mexico, Davila *et al.,* 2007^30^ worked with a sample of 50 patients, while in Chile, Abusleme *et al.,* in 2009 developed a study with 10 patients with periodontitis, seven with chronic periodontitis, and three with aggressive periodontitis. In Colombia, the study conducted in 2009 by Perez *et al.,* ^31^ was carried out with 15 patients, nine with chronic periodontitis, and six with aggressive periodontitis.In our country, studies are needed to permit identifying the most frequent *Porphyromonas* *gingivalis* *FimA* genotypes in healthy patients and in patients with different stages of periodontal disease with simple sizes calculated in such a manner that the results can be extrapolated to the Colombian population. Currently, the Periodontal Medicine Research Group of the School of Dentistry at Universidad del Valle is underway with a study which seeks to conduct genotipification of the *Porphyromonas gingivalis* *FimA* gene in healthy patients, with gingivitis and periodontitis from the city of Cali.

## Clinical importance in periodontal disease

*Porphyromonas gingivalis* is one of the main microorganisms implied in chronic and aggressive periodontitis. In Colombia, according to a multicentric study by Lafaurie *et al.,* in 2007^33^, prevalence of 71.5% was found of the microorganism in a sample of 325 patients with chronic periodontitis and 14.5% in a sample of 137 healthy patients and with gingivitis. The importance of identifying those more aggressive *Porphyromonas gingivalis* genotypes can lead in the future to developing new therapeutic approaches aimed at eliminating this pathogen, considered one of the main etiological factors of periodontal disease. Bearing in mind that this microorganism has a good ability to invade epithelial cells and fibroblasts, *in vitro* ^34^ ^,^ ^35^ the conventional scaling and planing mechanical therapy does not guarantee its total eradication; hence, it is recommended to complement this therapy with systemic antibiotics. However, in our environment the high intake of antibiotics and self-medication lead to high resistance to antibiotics, which influences on the result of these types of therapies^36^. Thereby, we may think of alternatives like immunoprophylaxis or immunotherapy by using target proteins important for the virulence of the microorganism, like type II and IV *Fimbriline*, considering these two as the most aggressive according to the results found in the studies previously referenced.

## Conclusions and recommendations

Bearing in mind that *Porphyromonas gingivalis* is one of the main microorganisms implied in chronic and aggressive periodontitis, and considering *Fimbriae* as one of its main virulence factors, it is important to recognize the different genotypes of the *FimA* gene and their distribution and prevalence in the world population. In the different studies reported in literature a distribution may be noted, where the *FimA* II genotype is the most frequently found in patients with periodontitis, while in healthy patients the most frequent genotype is *FimA* I. This could explain the existence of strains more virulent than others and because of this some patients can be positive for *P. gingivalis* and not develop periodontal disease. Few studies on *Porphyromonas gingivalis* *FimA* genotypes can be related to systemic complications attributed to the spread of bacteria via bacteremia.

We recommend conducting studies in Colombia to determine the frequency and distribution of the *Porphyromonas gingivalis* *FimA* genotypes in this population to determine the most virulent strains of such to carry out primary and secondary prevention and in the future perform research that uses target proteins like *FimB*riline for immunoprophylaxis or inmunotherapies that lead to eliminating this pathogen from subgingival microbiota.
